# Internet based patient education improves informed consent for elective orthopaedic surgery: a randomized controlled trial

**DOI:** 10.1186/s12891-015-0466-9

**Published:** 2015-02-07

**Authors:** Andrew Fraval, Janan Chandrananth, Yew M Chong, Phong Tran, Lillian S Coventry

**Affiliations:** Orthopaedic Department, Western Health, 160 Gordon St, Footscray, VIC 3011 Australia

## Abstract

**Background:**

Obtaining informed consent is an essential step in the surgical pathway. Providing adequate patient education to enable informed decision making is a continued challenge of contemporary surgical practice. This study investigates whether the use of a patient information website, to augment patient education and informed consent for elective orthopaedic procedures is an effective measure.

**Methods:**

A randomised controlled trial was conducted comparing the quality of informed consent provided by a standard discussion with the treating surgeon compared to augmentation of this discussion with an online education resource (www.orthoanswer.org). Participants were recruited from orthopaedic outpatient clinics. Patients undergoing five common orthopaedic procedures were eligible to participate in the trial. The primary outcome measure was knowledge about their operation. Satisfaction with their informed consent and anxiety relating to their operation were the secondary outcome measures.

**Results:**

There was a statistically significant increase in patient knowledge for the intervention arm as compared to the control arm (p < 0.01). Patients in the intervention arm, had an average score of 69.25% (SD 14.91) correct answers as compared to 47.38% (SD 17.77) in the control arm. Satisfaction was also improved in the intervention arm (p = 0.043). There was no statistically significant difference between the control and intervention arm relating to their anxiety scores (p = 0.195).

**Conclusions:**

The use of a patient education website as an augment to informed consent improves patient knowledge about their planned operation as well as satisfaction with the consent process whilst not increasing their anxiety levels. We recommend that all patients be directed to web based education tools to augment their consent.

**Trial registration:**

Australian New Zealand Clinical Trials Registry (ANZCTR) ACTRN12614001058662.

## Background

Obtaining informed consent is an essential step in the surgical pathway [1]. It assumes that the patient has a full understanding of the risks, perceived benefits, expected outcome, and alternative treatments for any given procedure [1]. Attaining a high quality informed consent is a complex process which requires information to be delivered to patients in a format that they can understand and retain, have the opportunity to reflect on and be able to respond with questions as well as express their opinion [1].

Patient education is at the core of obtaining adequate informed consent [2]. Consistent information delivered to the patient from the time they see their primary health care practitioner, to the time they give consent for a surgical procedure, is critical in aligning patients expectations of outcomes with the realities of what a certain treatment course can deliver. The challenge of providing adequate patient education, such that they are fully informed about their treatment options, is often undermined by long waiting lists within public health system for elective procedures. Patients often obtain information from friends and family or online rather than directly from medical practitioners [3]. This leads to a heterogeneous message about their condition and the treatment options suitable for them. These misconceptions may not be adequately overcome within the confines of a short surgical consultation in a busy outpatient clinic.

The importance of ensuring adequate patient education prior to surgical procedures is underpinned by the finding that a patient’s expectations for the outcome of a surgical procedure have a significant bearing on how satisfied they will be after their operation [4]. Thus an important part of optimising surgical outcomes relates to educating the patient adequately about the likely benefits and limitations of their proposed operation. Furthermore, a failure to provide adequate information relating to the risks or side effects of surgical procedures remains the leading cause of successful litigation against practicing surgeons [5]. Reports indicate that some patients receiving elective surgical procedures do not receive adequate information, the information is not fully understandable or the information patients receive is not tailored to their particular needs [6].

Given the demands of obtaining a high quality informed consent, various stradegies have been employed and investigated to assist the clinician to effectively inform their patients. Interventions previously employed include providing patients with written information in a paper or digital fromat, providing audiovisual presentations relating to their planned procedure, engaging in prolonged and structured discussions or testing patient’s knowledge following a discussion with their clinician [7]. Providing written additional information has been shown to be a useful intervention to improce informed consent. There may be a benefit to providing this information in an online rather than hard copy format due to it’s accessibility and the emergence of online patient information websites [7].

With this in mind, we have conducted a randomised controlled trial to answer the question: does exposing patients to an education website [8] as an augmentation to the standard consent process, improve the quality of informed consent attained. Our hypothesis is that we will find this intervention to be an effective tool in improving the quality of informed consent. This research builds on previous investigations which have shown positive outcomes following a range of consent augmentation strategies (Cohcrane). Our study is unique in that the intervention was carried out at a departmental level and across a number of elective Orthopaedic procedures.

## Methods

Between September 2013 to May 2014 a randomised controlled trial was conducted comparing the quality of informed consent provided by a standard discussion with the treating surgeon compared to augmentation of this discussion with an online education resource (www.orthoanswer.org). Ethics approval for this study was obtained from the Western Health low risk human research ethics panel.

### Participants and setting

Patients were recruited from the Western Health orthopaedic outpatient clinic. All patients that were booked for five common orthopaedic procedures were eligible to participate in the trial. The operations included were total knee arthroplasty, total hip arthroplasty, knee arthroscopy, shoulder arthroscopy and ACL reconstruction. Patients with an English reading level of grade 5 or below were excluded from participating. A patient’s reading level was assessed using the Rapid Estimate of Adult Literacy in Medicine (REALM) screening tool [9].

### Intervention

**Figure 1:**
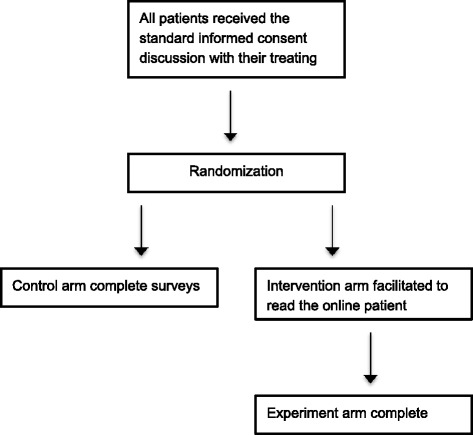
**Flow diagram of trial pathway.**

Computer randomisation was carried out by a random sequence generated by the STATA statistical software program using simple randomisation. Concealment to the randomisation sequence was by sealed envelope. Both control and intervention arms received the standard consent discussion as carried out by their treating surgeon. The control arm, completed surveys relating to the outcome measures of this study directly after their appointment with the surgeon. The experiment arm were facilitated to read the relevant section of the website, after having spoken to their surgeon about their planned operation. They were then directed to complete the same surveys (Figure 1).

Regarding the process by which the intervention was delivered, patients were facilitated by clerical staff employed by the hospital during this period. This occurred in conjunction with completion of standardised forms relating to the administrative process of being added to the elctive surgical wait list. The clerical staff were not part of the research team. Patients read the website whilst onsite utilising both desktop computers and tablet devices. The clerical staff were available to resolve any technical issues related to accesing the website. No clinical questions were answered by the clerical staff. No members of the research team were present during this period. Patients completed surveys immediately following having read the website. There were no measures in place to ensure that patients had completed reading the website other than a verbal acknowledgement to the clerical staff.

The orthopaedic units’ website (www.orthoanswer.org) was utilised as the patient education tool. It is written at a basic English reading level catering for patients with a reading level of grade 5 or above. It is designed as a walkthrough overview of each procedure along the lines of diagnosis and indications for surgery, complications, pre-operative, intra-operative and post operative care. This website is a free resource which has been developed by the Western Health Orthopaedic department. It has received no commercial funding and all contributers have done so on a purely voluntary basis. The website has been contributed to by orthopaedic residents and registrars, physiotherapists, occupational therapists and medical students. It has been reviewed by consultant orthopaedic surgeons from the Western health service to ensure that information is accurate and reflects current practice.

### Outcome measures

We assessed the quality of informed consent via three outcome measures. The primary outcome measure was knowledge about their operation. Satisfaction and anxiety were the secondary measures. Knowledge was assessed using an operation specific questionnaire, which tested patient’s understanding in the important areas relating to their ability to provide informed consent. These included the common side effects, uncommon but serious risks, length of the procedure, time to recovery following the operation, benefits of the operation and alternatives to surgery. Our surveys were based on the Deaconess Informed Comprehension Test, which is a validated survey to assess generalised knowledge of informed consent [10]. Satisfaction was assessed using the Client Satisfaction Questionnaire (CSQ-8). This is a validated eight-question survey to assess satisfaction with a service provision [11]. Anxiety was assessed using the State-Trait Anxiety Index, which is a validated survey to assess a patient’s level of anxiety, in this case in relation to their planned surgery [12].

### Demographics

Demographic data collected included gender, age and their highest level of education attained. Education level was recorded in the categories of: no formal education, primary school, secondary school, trade or Tafe certificate, tertiary education and postgraduate education.

### Statistics

Results were analysed with the STATA statistical software package. Continuous data such as survey scores and age of patients is reported in terms of the mean and 95% confidence interval. The mean of continuous data between intervention arms is compared using the T-test. Dichotomous data such as education is compared using z-test of two proportions. Gender is compared using the chi-square test.

To detect an improvement of 20% in the primary outcome measure of knowledge in the experiment group, which is in keeping with the observed magnitude of similar trials conducted previously [13,14], with a two-sided 5% significance level and a power of 80%, a sample size of 91 patients per group was necessary.

## Results

**Figure 2:**
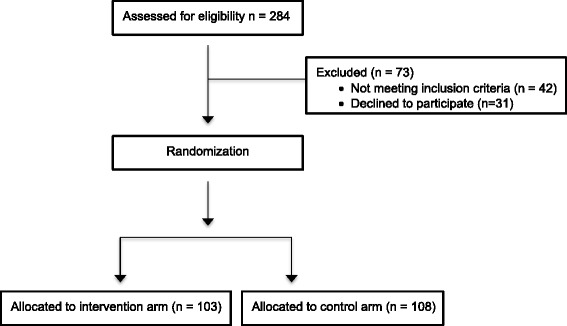
**Flow diagram of the progress of the participants through trial pathways.**

284 patients were assessed for eligibility. 42 patients were excluded due to not reaching the reading standard necessary to participate in the trial. 31 patients declined to participate in the study. 211 patients were randomised to participate in the trial. Randomisation resulted in 103 patients being allocated to the intervention arm and 108 to the control arm (Figure 2).

The average age of patients was 54.29 years in the intervention arm and 53.70 in the control arm. There was no clinically significant difference between the demographics of the two groups. Education level was distributed in a similar pattern between control and intervention arms with secondary school education being the most common level of education. There were no clinically significant differences between the education levels of the control and intervention arms. There was also no clinically significant differences in the distribution of operation received between the two arms of the study.

There was a clinically significant increase in patient knowledge for the intervention arm as compared to the control arm (p < 0.01). Patients in the intervention arm, had an average score of 69.25% correct answers as compared to 47.38% in the control arm (Table 1).

**Table 1:** **Results for primary and secondary outcome measures**

	**Internet (n = 103)**	**Control (n = 108)**	**P value**
**Outcome measures**			
Knowledge (SD)	69.25% (14.91)	47.38% (17.77)	< 0.01^a^
Satisfaction (SD) (maximum score = 24)	20.59 (2.34)	19.71 (3.76)	0.043^a^
Anxiety (SD) (maximum score = 84)	36.75 (12.19)	38.98 (12.70)	0.195^a^

^a^Independent samples – t test.

Satisfaction was also improved with a score of 20.59 in the intervention arm vs 19.71 in the control arm although not to the same significance level as for patient knowledge (p = 0.043). There was no difference between the control and intervention arm relating to their anxiety scores (p = 0.195).

## Discussion

Our study has shown that patient knowledge was improved by the use of a website designed specifically for patient education.. Exposing patients to further information relating to the risks associated with a procedure did not increase their anxiety or fear of the operation. These results are consistent with previous studies which have shown consent augmentation through the use of written and online information to be an effective measure [1]. Multiple approaches have been utilised to improve informed consent for patients undergoing surgical procedures. The use of written information has been previously investigated and found to be beneficial [8]. This study builds on these findings and provides evidence that an online format is also an effective method of improving informed consent.

Over half the patient’s attending the Western Health orthopaedic outpatient clinic already access the internet for information relating to their condition [15]. Unfortunately, websites frequently encountered via search engines are often not written at the appropriate reading level, of poor quality or financially biased [16-18]. This undermines the use of patient directed online education and re-enforces the need for clinicians to be providing their patients with high quality online education resources.

Our study is the first RCT to examine the efficacy of a free online patient education tool designed for patients in a public hospital setting, providing consent augmentation for multiple surgical procedures conducted at a departmental level. Free online resources have the potential benefit of being a readily accessible resource available to general practitioners, surgeons and patients. This may allow for early referral to the website by primary healthcare practitioners before patients attend their specialist surgical outpatient appointment. This practice could facilitate reinforcement of consistent information throughout the surgical pathway from referral to completion of the procedure. It may also allow patients to formulate informed questions to be clarified at their specialist appointment. This is a potential area of future research in the area of online patient education relating to orthoapedic surgical procedures.

When eliciting informed consent, clinicians tend to focus on communicating the specific technical risks relating to the planned procedure [19]. Whilst these risks may be of shared interest between surgeon and patient, other factors relating to consequences of a procedure such as pain, length of stay in hospital and time off work after the operation are often overlooked. This leaves many patients with the opinion that the primary function of the consent form is to protect the hospital, rather than prepare the patient for the operation [20]. Patients are often left to discover their post-operative course in the days following their procedure rather than at the time of considering whether to proceed with the operation. Adequate information provision has broad benefits for patients, including increased satisfaction, reduced emotional distress, and reduced use of analgesia underlining the importance of effective measures to improve patient education [1]. It is possible that by directing patients to online resources both before and after being consented for elective orthopaedic procedures, some of these difficulties with preparing patients for their operations may be overcome. This however is not specifically addressed by our research and would be a potential area of focus for future research in this area.

The main limitation of this study is the lack of longitudinal follow-up to examine whether the improvement in knowledge is sustained. Previous studies have investigated longitudinal follow up of similar consent augmentation interventions and found that an improvement of knowledge at the time of intervention did have a longitudinal measured effect [3,13]. However there are heterogeneous results in this area, with other trials failing to show persistence of improvements [21]. Given the heterogeneous nature of results in this area, further research could be carried out to determine the persistence of the observed effect on patient education using this intervention.

## Conclusions

In conclusion we have shown that the use of a patient education website improves patient knowledge about their planned operation as well as satisfaction with the consent process. Exposing patients to additional web based information did not affect their anxiety levels relating to their planned operation. When considering possible methods of consent augmentation, online patient education tools should be considered as a viable option.
