# Erratum to: Internet based patient education improves informed consent for elective orthopaedic surgery: a randomized controlled trial

**DOI:** 10.1186/s12891-015-0660-9

**Published:** 2015-10-06

**Authors:** Andrew Fraval, Janan Chandrananth, Yew M. Chong, Lillian S. Coventry, Phong Tran

**Affiliations:** Orthopaedic Department, Western Health, 160 Gordon St, Footscray, VIC 3011 Australia

## ERRATUM

The original version of this article unfortunately contained a mistake. The sequence of the author names was incorrect in the HTML and PDF versions of this article. The correct author list should read:

Andrew Fraval*^†^, Janan Chandrananth^†^, Yew M Chong^†^, Lillian S Coventry^†^ and Phong Tran^†^
